# UV-protective sunscreen compounds and their differential accumulation across ocular tissues in fish eyes from Baltic Sea

**DOI:** 10.1038/s41598-026-68431-8

**Published:** 2026-08-27

**Authors:** Ulf Karsten, Laureen Walberg, Uwe Krumme, Friederike Schaub, Oliver Stachs, Karsten Sperlich, Tobias Lindner, Fabian Hammerle, Johanna M. Gostner, Cornelia A. Karg, Markus Ganzera

**Affiliations:** 1 Department of Applied Ecology and Phycology, Institute of Biological Sciences, University of Rostock, Albert-Einstein-Strasse 3, 18051 Rostock, Germany; 2 Interdisciplinary Faculty, Department of Maritime Systems, University of Rostock, Albert-Einstein-Strasse 21, 18051 Rostock, Germany; 3 Thuenen Institute of Baltic Sea Fisheries, Alter Hafen Süd 2, 18069 Rostock, Germany; 4 Department of Ophthalmology, Rostock University Medical Center, Doberaner Straße 140, 18057 Rostock, Germany; 5 Interdisciplinary Faculty, Department Life, Light & Matter, University of Rostock, Albert-Einstein Str. 25, 18051 Rostock, Germany; 6 Core Facility Multimodal Small Animal Imaging, University Medical Center Rostock, 18057 Rostock, Germany; 7 Institute of Diagnostic and Interventional Radiology, Pediatric Radiology and Neuroradiology, University Medical Center Rostock, 18057 Rostock, Germany; 8 Institute of Pharmacy, Pharmacognosy, University of Innsbruck, Innrain 80-82, 6020 Innsbruck, Austria; 9 Institute of Medical Biochemistry, Medical University of Innsbruck, 6020 Innsbruck, Austria; 10 Core Facility Metabolomics II, Medical University of Innsbruck, 6020 Innsbruck, Austria

**Keywords:** Demersal fishes, Mycosporine-like amino acids, Ocular tissues, Piscine predators, Trophic transfer, Visual ecology, Ecology, Ecology, Zoology

## Abstract

Many fish species achieve ultraviolet radiation (UVR) protection by accumulating mycosporine-like amino acids (MAAs) from their diet. High concentrations are often found in the eyes, but the localization of MAAs in specific fish eye tissues is unknown. In the present study we dissected the eyes of six fish species, and prepared cornea, lens, vitreous body and a mixed tissue sample consisting of retina, choroidea and sclera (RCS). Furthermore, to test if MAAs are transferred further up the food web, we investigated the occurrence of MAAs also in eyes of warm-blooded piscine predators. Most, but not all, fish eye tissue samples contained the MAAs palythine, asterina-330, usujirene and palythene, but in different quantitative patterns. The highest MAA concentrations were found in the cornea, lens and vitreous body, while the innermost RCS layer showed a much smaller content. All data indicate that most MAAs are mainly located in the cornea and/or lens and vitreous body, thereby providing an improved photoprotection of the RCS layer, and in particular of the innermost, UVR-sensitive retina. No MAAs were detected in the eyes of cormorants and piscivorous mammals, suggesting that the trophic transfer of MAAs from the primary producers via zooplankton apparently ends in the fish.

**Supplementary Information:**

The online version contains supplementary material available at 10.1038/s41598-026-68431-8.

## Introduction

Solar radiation is essential for aquatic and terrestrial primary production, and it is the base for higher trophic levels in the respective food webs^1^. However, the ultraviolet (UV) part has such high photon energy that essential biomolecules can be potentially negatively affected^2^. The main vulnerable and ubiquitous target biomolecules are DNA and proteins, which are photochemically damaged or even destroyed after UV absorption^2^. Therefore, ultraviolet radiation (UVR) is a mutagenic agent and hence considered as a strong evolutionary selective force^3^.

In the marine realm UVR is also biologically harmful to all types of marine organisms, from bacteria to vertebrates such as fish^4^. However, UVR is influenced by an array of meteorological, oceanological and geographical factors, including geolocation, depth in the water column, sun angle, season, clouds, waves, tidal currents and optical properties of the water. Typically, off-shore water masses exhibit a high transparency and hence rather deep penetration of incident UVR, while coastal waters are often characterized by suspended inorganic and organic particles along with so-called yellow humic substances (chromophoric dissolved organic matter, cDOM) which strongly attenuate UVR penetration into the water column^5^. Consequently and depending on the environmental conditions, underwater UVR can be highly variable in space and time.

UVR can have a detrimental influence on fish, impacting their development, growth, physiology, behavior, and immune systems. These effects can range from skin damage and reduced growth rates to increased susceptibility to diseases up to mortality^6,7^. In their comprehensive review Alves and Agusti (2020)^7^ indicated that the developmental stage of fish is the key to understand population fitness and survival under UVR stress. Larvae exposed to UVR often exhibit higher mortality rates as well as an enhanced number of developmental malformations compared to juveniles/adults, with the skin and gills being the most affected tissues^7^. In addition, the juvenile and adult stages of many fish species show a conspicuous decline in growth along with adverse behavioral, physiological, and biochemical changes upon UVR stress. The underlying molecular and cellular processes are often impaired as reflected in raising incidences of DNA damage, apoptosis and changes in the antioxidant status of different tissues^8^.

In response to the adverse effects that UVR can exert on fish, they have developed various protective strategies to escape or to cope with this stressor. Well documented are behavioral changes to avoid UVR exposure, for example, twilight or nocturnal activity, use of shaded places or greater depths^8,9^. Other photo-protective strategies include physico-chemical barriers such as scales or epidermal mucus, efficient DNA repair mechanisms (e.g., by photolyase), recycling of damaged protein or detoxification of reactive oxygen species (ROS), as well as the accumulation of UV-sunscreen compounds^7^. Nevertheless, since only a relatively small number of more than 34,000 fish species on our planet (www.fishbase.org; access date 14th April 2026^10^) has been investigated for UVR effects, it is likely that the known mechanisms are by far not exhaustive^7^.

The best studied UV-sunscreens in marine organisms are the so-called mycosporines and mycosporine-like amino acids (MAAs), which include > 80 chemically similar structures^2,11^, that occur in cyanobacteria, different algal groups, invertebrates, and fish^11,12^. MAAs represent organic molecules with molecular weights below 400 Da and interesting physico-chemical properties. The most important feature is their hydrophilicity along with their very high molar extinction coefficients (Ԑ = 28,100 to 50,000 M^− 1^ cm^− 1^) in the UV range. The chemical structures of MAAs have been elucidated after isolation and purification from biological samples followed by liquid chromatography and mass spectrometry (LC-MS) and nuclear magnetic resonance spectroscopy (NMR) techniques^13^. The conjugated molecule structure consists of a cyclohexenone (mycosporines) or cyclohexenimine (MAAs) core that is substituted with an amino acid or its imino alcohol. Some widely distributed representatives, such as porphyra-334 or shinorine, are chemically stable under heat and irradiation exposure^2^, thereby supporting their UV-sunscreen function^14,15^. In addition, MAAs may also act as antioxidants and are involved in stress responses^11,16^.

Most of the reported MAAs have their biological origin in the primary producers, i.e., different groups of phytoplankton (e.g., diatoms, dinoflagellates etc.) and seaweeds (e.g., red algae)^11^. The shikimate pathway was experimentally proven as the underlying biochemical mechanism, although alternative biosynthetic routes might exist in different taxa (for review see^17^). However, MAAs were also found in many marine invertebrates like corals or sea urchins, as well as in numerous fish species^12,16^. To the best of our knowledge, fish acquire MAAs from their diet, pointing to a trophic transfer from the primary producers via the food web to higher trophic levels^12^. If MAAs are transferred through the food web and provide UV protection for vertebrates like fish, it is of course relevant to assess whether MAAs are also found in the predators of fish or not. However, the trophic fate of MAAs in warm-blooded piscine predators like cormorants, grey seals or otters is unknown.

Bonin and coworkers^12^ investigated the occurrence of MAAs in the dissected eyes of 39 fish species from the northern Atlantic and found aplysiapalythine A, asterina-330, palythene, palythine, porphyra-334, shinorine and usujirene in almost all samples. Total MAA values ranged from trace amounts up to > 4.2 mg g^− 1^ dry weight, with the highest concentrations estimated in *Sprattus sprattus*^12^. In a follow up study, Hammerle et al. (2025)^18^ examined the qualitative and quantitative distribution of MAAs in the organs of European flounder (*Platichthys flesus*), European plaice (*Pleuronectes platessa*), and turbot (*Scophthalmus maximus*), and found that the highest MAA concentrations occurred in the eyes, but minor values also in other organs. These data confirmed previous investigations on warm-temperate to tropical fish species, which contained MAAs primarily located in the eyes as well^19,20^. However, previous studies did not distinguish between different tissue types in the eyes, except for Thorpe et al.^21^ who reported the occurrence of MAAs mainly in the lens of fish eyes. They are anatomically similar to those of mammals, but have a more spherical lens. Light enters the fish eye through the cornea, passing pupil, lens, and vitreous body before reaching the retina^22^. Whether MAAs are indeed primarily located in the lens or also occur in other tissues of fish eyes is unknown.

This study focused on the localization of MAAs in different compartments inside the fish eye. Samples of six fish species, caught in the western Baltic Sea, were dissected into cornea, lens, vitreous body, and a mixed sample consisting of retina, choroidea and sclera. The different tissues were freeze-dried, extracted, and qualitatively and quantitatively analyzed for their MAA composition using high performance liquid chromatography with diode array detector (HPLC-DAD). We hypothesized the highest MAA levels in the cornea, which is the outermost layer of the eye and hence the most UVR exposed tissue of this organ. Additionally, to preliminarily evaluate if MAAs are further transferred to the eyes of fish-feeding warm-blooded predators, we report results from first measurements of MAAs in eyes of major piscine predators from the temperate region like cormorants, and marine and freshwater mammals.

## Materials and methods

### Fish samples, eye dissection and tissue preparation

**Fig. 1:**
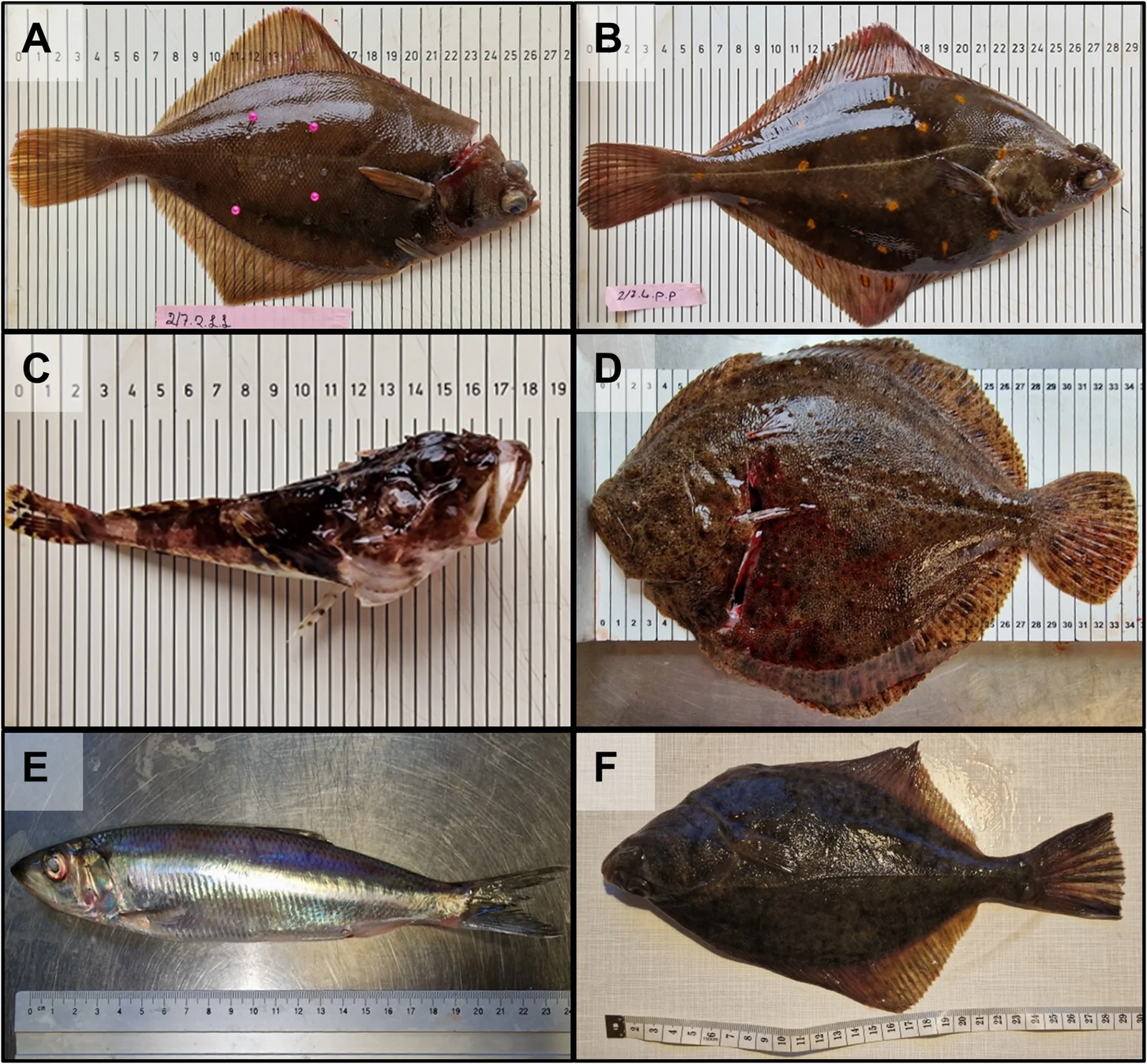
Pictures of the six investigated fish species caught by demersal trawl in June and July 2024 from the western Baltic Sea. A: dab (*Limanda limanda*); B: European plaice (*Pleuronectes platessa*); C: sculpin (*Myoxocephalus scorpius*); D: turbot (*Scophthalmus maximus*); E: herring (*Clupea harengus*); F: European flounder (*Platichthys flesus*).

In this study adults (5–7 replicates) of six abundant fish species of the western Baltic Sea (Fig. 1) were analysed, the European flounder (*Platichthys flesus*, total length range: 28–31 cm, 6 replicate animals), European plaice (*Pleuronectes platessa*, total length range: 27–28 cm, 5 replicate animals), turbot (*Scophthalmus maximus*, total length range: 32–34 cm, 5 replicate animals), common dab (*Limanda limanda*, total length range: 25–27 cm, 6 replicate animals), shorthorn sculpin (*Myoxocephalus scorpius*, total length range: 17–19 cm, 5 replicate animals), and herring (*Clupea harengus*, total length range: 23–25 cm, 7 replicate animals) (Fig. 1).

The national state-funded Thünen Institute of Baltic Sea Fisheries in Rostock has governmental functions, monitors fish stocks and develops environmentally friendly fishing techniques to reduce bycatch. Therefore, the Thünen Institute adheres to strict ethical and legal principles regarding animal welfare. It is bound by the German Animal Welfare Act as well as European directives. The guiding principle here is the ethical principle of the “3Rs”: Replace, Reduce, and Refine. The Thünen Institute does not legally need its own ethical commission but rather appoints its own animal welfare officer, who monitors compliance with the Animal Welfare Act during scientific projects directly on-site. The competent state regulatory authority for animal welfare and animal ethics at the Thünen Institute of Baltic Sea Fisheries in Rostock is the Veterinary and Food Control Office (VLÜA: Veterinär- und Lebensmittelüberwachungsamt) of the City of Rostock, and at the federal state level, the State Office for Agriculture, Food Safety, and Fisheries of Mecklenburg-Western Pomerania (LALLF: Landesamt für Landwirtschaft, Lebensmittelsicherheit und Fischerei Mecklenburg-Vorpommern). The fish were not caught specifically for the present study, but rather as part of a government-mandated monitoring cruise, and all experimental protocols followed the ethical and legal principles regarding animal welfare. All fish species were caught by demersal trawl in June and July 2024 in shallow coastal waters of the western Baltic Sea at depths < 25 m. The capture, handling and killing of the fish were conducted in strict accordance with the institutional, national, and international guidelines for the ethical treatment of animals in research. Anaesthetics or analgesics were not used. The fish were percussion stunned with a single blow to the head and killed by cardiac puncture to ensure a rapid and reliable death. This procedure was carried out by experienced personnel trained to immediately induce unconsciousness.

Flounder, plaice and dab are benthivorous, feeding on bivalves and other invertebrates. **S**horthorn sculpin also feeds on a variety of invertebrates. Turbot is a piscivorous flatfish species. Herring mainly feeds on planktonic zooplankton. All species occur in shallow coastal waters, especially from spring to autumn when natural insolation is usually high.

The freshly caught fish (5–7 specimens per species) were percussion stunned by a blow to the head, killed by a cut through the spine and kept on ice prior to dissection of the eyes. To allow access to the eyes, the jaw and gill cover were cut with bone scissors and the skull was cut through to the eye socket using binocular microscope, scalpel, and forceps. Once access to the eye was gained, the skin around each eye was cut, and the eye lifted with forceps, allowing the optic nerve to be easily severed. Afterwards, the eye was removed from the eye socket and the remaining fatty tissue removed using scissors and forceps.

The freshly prepared fish eyes were kept on ice prior to investigation with Magnetic Resonance Imaging (MRI) as a non-invasive imaging technology that produces three dimensional detailed anatomical images. MRI was conducted on a 7 T MRI scanner (BioSpec 70/30, Bruker Biospin MRI GmbH, Ettlingen, Germany) equipped with a BGA-12 S HP gradient (bore size of 11 cm) and a circularly polarized volume coil (rat body coil with 112 mm/72 mm outer/inner diameter, Bruker Biospin). First, a fast T2-weighted (T2w) was acquired. Following the localizer, a high-resolution T2w turbo spin-echo sequence (TurboRARE – Rapid Acquisition with Relaxation Enhancement) of the eye was performed in all planes. Imaging parameters were time of echo (TE)/time of repetition (TR): TE/TR (sagittal) = 28.71/2978, TE/TR (coronal) = 28.71/2500, TE/TR (axial) = 26.93/2669; matrix size 250 × 200; FOV = 40 mm × 40 mm; in-plane resolution 0.1 mm × 0.1 mm; slice thickness 0.8 mm; time of acquisition: 09:55 min (sagittal), 11:00 min (coronal), and 8.53 min (axial).

Additionally, Optical Coherence Tomography (OCT) was employed, a non-invasive, optical imaging modality generating cross-sectional tissue images. This technique is widely used in ophthalmology to assess the anterior and posterior segment of the eye, especially the cornea and retina. OCT Imaging of fish eyes was performed using an investigational research device named SPECTRALIS HighRes OCT (Heidelberg Engineering GmbH, Heidelberg, Germany). It was equipped with a prototypic high-resolution anterior segment objective lens. The device utilizes a superluminescent diode for OCT at a central wavelength of 839 nm with 141 nm spectral bandwidth and features an extended reference arm to enable the prototypic objective lens^23^. The lateral and axial resolutions were determined to be 3.5 μm and 3.2 μm by imaging a USAF resolution target and by calculating the normalized coherence function, respectively. Scan parameters such as B-scan size, number of A-scans per B-scan, and image averaging (ART) were adjusted to optimize visualization of corneal structures and the lens.

After the MRI and OCT measurements of the intact eyes, the different ocular tissues were dissected. The individual layers of the cornea were gently lifted and removed with eye forceps, after separation at the corneal limbus with eye surgery scissors. This allowed the lens and the vitreous body (corpus vitreum) to be removed with eye forceps. The remaining tissue consisted of the retina, choroidea and sclera, which could not be further separated and hence were considered as one sample. If the time between eye removal and tissue dissection took more than 1 h, the eyes were transferred to 0.9% aqueous NaCl solution and stored in a refrigerator at 8 °C until further preparations.

After total dissection, the different eye tissues (cornea, lens, vitreous body and retina/choroidea/sclera) were briefly washed with distilled water to remove external tissue remains, frozen at −18 °C and afterwards freeze-dried in a lyophilizer (Alpha 1–4 LSCPlus, Martin Christ GmbH, Osterode am Harz, Germany). The freeze-dried tissues were stored at −18 °C prior to MAA extraction.

### Eyes of piscine predators

It is currently not known if MAAs are also transferred via the food web into the eyes of mammals and other vertebrates that feed on fish. Therefore, the possible presence of MAAs in the eyes of typical piscine predators of the temperate zone of Western Europe was assessed. Eyes of cormorants (*Phalacrocorax carbo*) were provided by the Museum der Westlausitz Kamenz (Department of Zoology), while eyes of harbor porpoise (*Phocoena phocoena*), harbor seal (*Phoca vitulina*), grey seal (*Halichoerus grypus*), North American racoon (*Procyon lotor*) and otter (*Lutra lutra*) were provided by the Institute for Terrestrial and Aquatic Wildlife Research, Büsum, Germany (University of Veterinary Medicine Hannover Foundation). None of these fish predators were killed for any of the eye samples mentioned. All animals were found dead and were promptly dissected by the institutions mentioned; various tissues, including the eyes, were frozen for later analysis. All predator eyes were sent as frozen samples via express courier to the University of Rostock, and stored at −18 °C prior to MAA extraction.

### Extraction of MAAs

After freeze-drying, the different tissues were weighed, to reach 5–10 mg dry weight per mL extraction solvent, which is the optimum ratio for extracting MAAs from fish eyes^12^. To do so, each weighed tissue sample was transferred to a screw-capped vial and 1 mL of aqueous methanol v/v (25%) was added. The vials were then vortexed and placed in a 45 °C water bath for 3–4 h. After 2 h and at the end of the extraction time, the samples were vortexed again. Afterwards, the vials were centrifuged for 5 min (16,058 x *g*) (Biofuge pico, Hereaus, Hanau, Germany). 800 µL of the supernatant was transferred to a new vial and evaporated overnight to dryness using a rotational vacuum concentrator (Christ RVC 2–25, Martin Christ GmbH, Osterode am Harz, Germany). The dried pellets were re-dissolved in 800 µL of HPLC-grade water, vortexed for 30 s, and centrifuged for an additional 5 min (16,058 x *g*) (Biofuge pico, Heraeus, Hanau, Germany). The supernatants were then transferred into HPLC vials and stored in a freezer at −18 °C until being analyzed. In case of smaller tissue amounts (< 1 mg) the volume of the extraction solvent remained the same, but the re-dissolution HPLC water volume was reduced to 200 and 400 µL, respectively. Finally, the aqueous supernatants were transferred to HPLC vials after passing through a 0.45 μm cotton filter (WhatmanTM, Germany) prior injection onto the HPLC column.

### Qualitative and quantitative MAA analysis

Analyses were performed on an Agilent 1220 Infinity II HPLC system (Agilent Technologies Deutschland GmbH & Co. KG, Waldbronn, Germany) equipped with an isocratic pump, autosampler, column thermostat, and diode-array detector (set at 330 nm, range: 280–400 nm). A Phenomenex Synergie Fusion RP-18 column (4 μm, 250 × 3.0 mm) (Phenomenex, Aschaffenburg, Germany), protected by an RP-18 guard cartridge of the same material (4 × 3 mm I.D.), was used to separate the MAAs. The flow rate of the eluent, 2.5% of methanol in water (v/v) plus 0.1% acetic acid (v/v), was 0.5 mL min^− 1^. The temperature of the column oven was set to 30 °C, and the injection volume was 10 µL. Biological standards from known organisms were used for identification and quantification: mycosporine-glycine (λ_Max_ = 310 nm, marine lichen *Lichina pygmaea*), shinorine (λ_Max_ = 320 nm, red alga *Mastocarpus stellatus*), asterina-330 (λ_Max_ = 330 nm, eyes of *Plectropomus leopardus*), porphyra-334 (λ_Max_ = 334 nm, red alga *Porphyra umbilicalis*) and palythine (λ_Max_ = 320 nm, eyes of *Sprattus sprattus*) (see Fig. S1). These biological standards were calibrated using chemically pure stock solutions of shinorine, palythine, asterina-330, and porphyra-334, which were available from a previous study^24^. Sample chromatograms of the different eye tissues were compared with those from the biological standards to identify MAAs based on matching retention time and absorbance spectrum. The area under each integrated peak of the samples and reference concentrations of MAAs in their biological standards were used to quantify MAAs in the different tissues (mg g^–1^ dry weight).

Aqueous stock solutions of palythine, asterina-330, and aplysiapalythine A originating from our previous study^12^ were used for calibration, and the appropriate calibration curves were created by plotting the peak areas versus the MAA concentrations. The regression parameters were calculated by linear regression analysis using Microsoft Excel. The limit of detection (LOD) and limit of quantification (LOQ) for each MAA were calculated from the regression models, and % recovery, and linear range determined (Table 1). Precision could not be determined due to the small sample size of only up to 10 mg per tissue. The calibration curve of aplysiapalythine A had to be applied for usujirene and palythene quantification since no standards are available.

**Table 1:** HPLC validation parameters for the quantified MAAs detected at 330 nm in the present study. Calibration data of Aplysiapalythine A were used for the quantification of usujirene and palythene.

Parameter	Palythine	Asterina-330	Aplysiapalythine A
Regression equation	y = 139,440 x + 16,461	y = 196,159 x – 4344	y = 149,303 x + 1629
Coefficient of determination	R^2^ = 0.9997	R^2^ = 0.9992	R^2^ = 0.9997
Linear range (µg mL^− 1^)	1–200	1–200	1–200
Limit of detection (µg mL^− 1^)	0.07	0.15	0.05
Limit of quantification (µg mL^− 1^)	0.20	0.46	0.16
Percent recovery	92.3	89.8	-

The applied HPLC method is a compromise between speed (it is fast, allowing a high sample throughput) and separation efficiency (the isomers usujirene (cis-form) and palythene (trans-form) could not be fully resolved). In the Bonin paper^12^ we used a slightly different column and a gradient eluent which resulted in complete separation of both chemically similar MAAs, but led to a very long run and column recalibration time. We compared the results of a total eye extract of herring with both HPLC methods and found high agreement in all quantitative values.

### Statistics

For all ocular tissues, 5 to 7 replicate eye samples (from 5 to 7 individual fish species) were analyzed. Mean values ± standard deviations were calculated using Excel version 2025 (Microsoft Corporation, Redmond, USA). Mean values were compared using Kruskal-Wallis tests. When significant differences were found (*p* < 0.05), Tukey’s post hoc tests were applied. The statistical analyses were performed in *R* (version 4.4.2, R Core Team, 2024) using RStudio.

## Results

### Fish eye dissection and anatomy

**Fig. 2:**
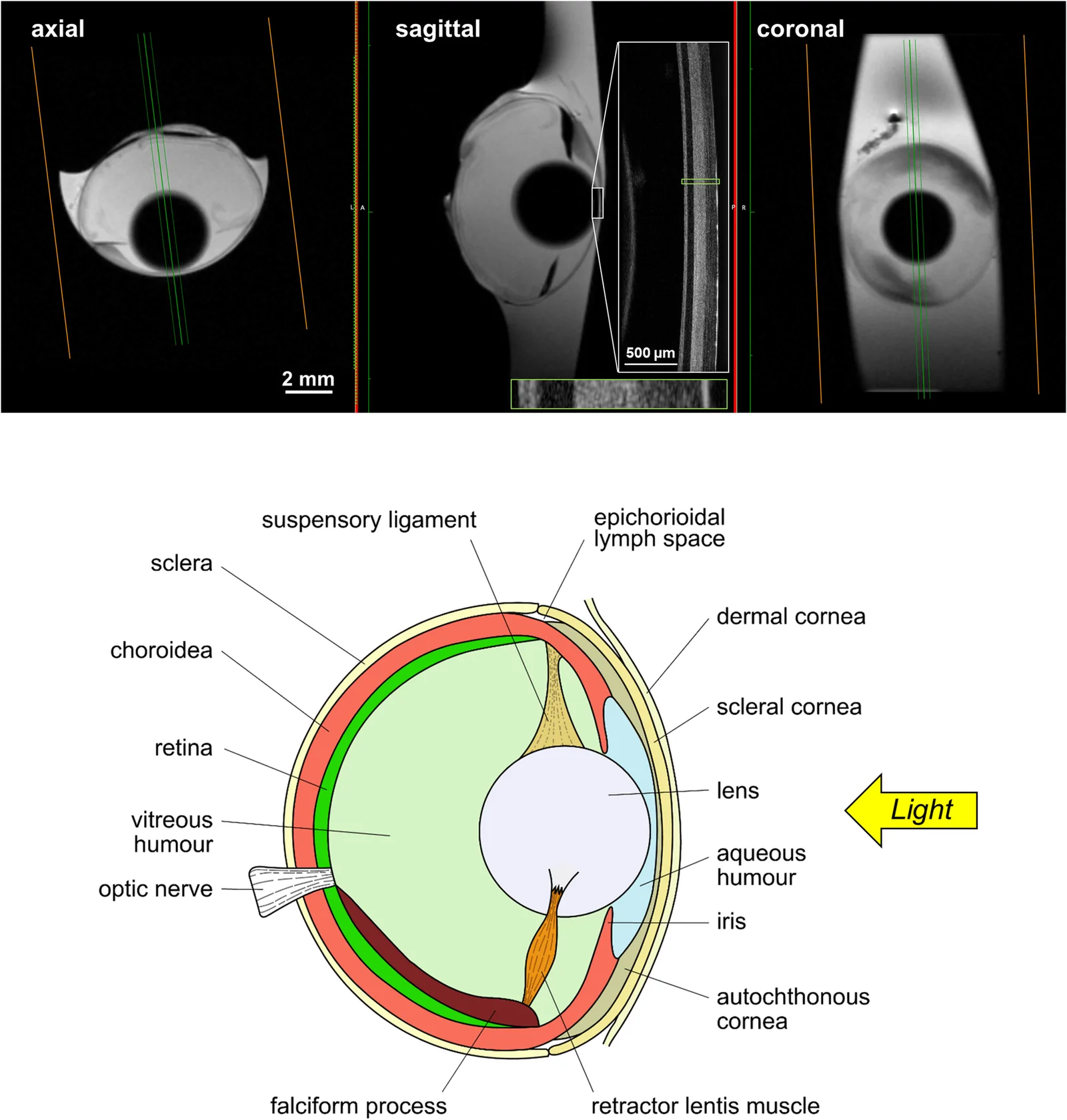
Magnetic Resonance (MRI) images of a fish eye prepared from *Clupea harengus* in all planes, i.e. axial, sagittal and coronal, indicating the main anatomical features. Optical Coherence Tomography (OCT) measurements of the cornea (inset sagittal) were also performed, showing the different tissue layers. Schematic cross-cutting view of the *Clupea harengus* eye based on the MRI and OCT data (adapted from Gretarsson 2019). Bony fish eye [SVG]. Wikimedia Commons. https://commons.wikimedia.org/wiki/File:Bony_fish_eye_multilang.svg. CC BY-SA 4.0). Light enters the eye through the cornea (3 different layers), passing lens, pupil and vitreous body before reaching the retina together with the associated tissue layers choroidea and sclera.

Dissection of eyes and their different ocular tissues from the six investigated fish species was feasible using specific eye forceps and eye surgery scissors, which are typically applied as micro-tools in human cornea transplantations. Therefore, it was possible to clearly separate the cornea, lens and vitreous body, while the innermost tissues consisting of the retina, choroidea and sclera could not be further dissected and separated because of their small size (Fig. 2). The latter three tissues were combined as one sample due to practical reasons.

High-resolution T2-weighted TurboRARE images of ex-vivo fish eyes provided clear anatomical delineation in axial, sagittal, and coronal planes (representative example: *Clupea harengus)*. The vitreous body appeared uniformly hyperintense, while the compact spherical lens was distinctly hypointense with sharp boundaries. The cornea formed a thin, relatively low-signal rim at the globe periphery, and the inner coats (RCS) were visualized as a continuous band facing the vitreous, without sufficient contrast to separate the three corneal layers (dermal cornea, scleral cornea and autochthonous cornea) individually at the achieved resolution (see Fig. 2). In contrast, high-resolution anterior segment OCT (see white-framed inset in middle panel) reliably resolved even the corneal microanatomy (cf. magnified green-framed inset in middle panel). According to Collin & Collin (2001)^25^ the corneal sub-layers of *Clupea harengus* are from anterior to posterior: the epithelium and the dermal stroma as parts of the dermal cornea; the mucoid layer; and the anterior scleral and posterior scleral stroma with the endothelium being parts of the scleral cornea. The autochthonous layer is rare in teleosts^25,26^ and was not observed in the OCT images. The epithelium appeared as a thin layer (13 μm) together with a surface reflection. The dermal stroma (97 μm thick) appeared darker (hyporeflective) as the adjacent mucoid layer (108 μm), while the anterior stroma (57 μm thick) was darker again. The leftmost layer (27 μm) could not be resolved in detail but should consist of iridescent layer, posterior scleral stroma, Descemet’s layer and the endothelium. Where the scan intersected the anterior lens, the anterior lens capsule appeared as a bright, curved line. The OCT panel (inset) alongside the MRI series in Fig. 2 illustrates the layer-by-layer correspondence between modalities.

### Qualitative and quantitative MAA composition across ocular tissues of fish eye

**Fig. 3:**
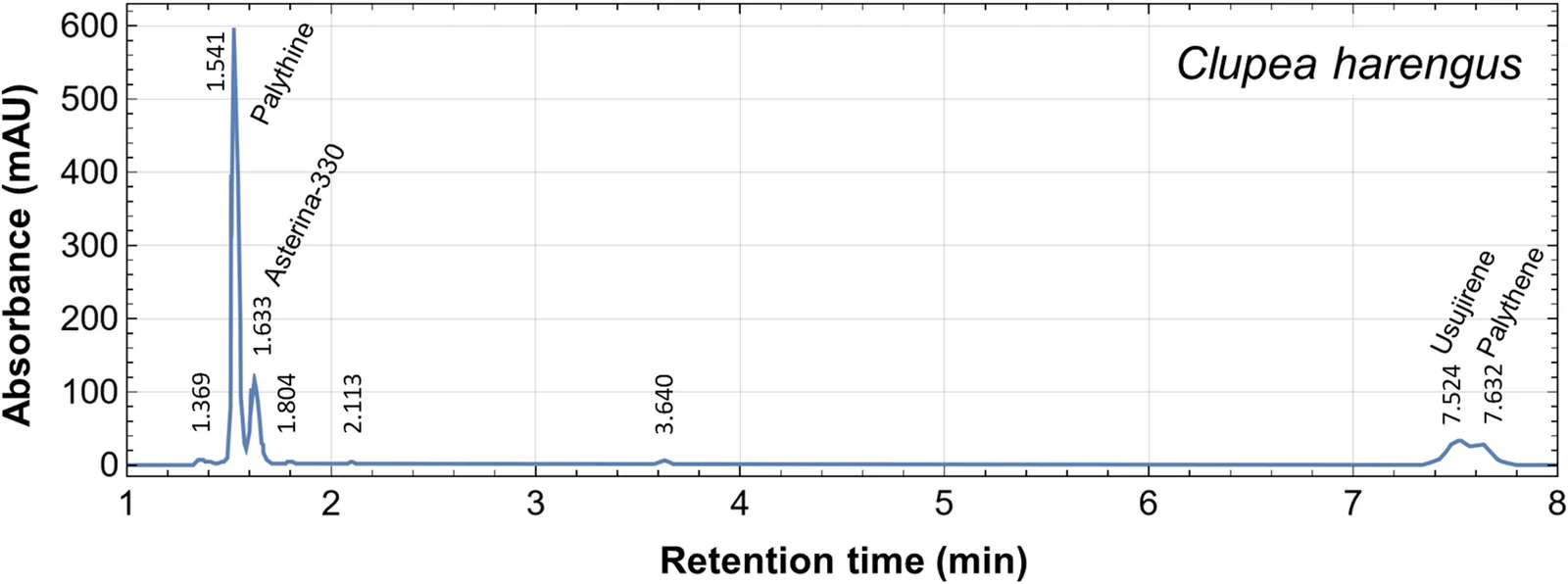
Representative HPLC chromatogram of a methanolic extract of a lens prepared from the eye of herring, *Clupea harengus*. The diode-array detector was set at 330 nm (range: 280–400 nm) and the flow rate of the eluent was 0.5 mL min^− 1^ (for analytical details and settings see Material and Methods). The peaks represent individual MAAs with different retention times: 1.54 min – palythine; 1.63 min – asterina-330; 7.52 min – usujirene; 7.63 min – palythene. The remaining very small peaks could not be identified. See also chromatograms of standard MAAs (Fig. S1).

The applied analytical HPLC method revealed that the MAAs palythine and asterina-330 exhibited retention times of 1.54 and 1.63 min; both could be well separated (Fig. 3, Fig. S1). The other MAAs usujirene and palythene showed a retention time of 7.52 and 7.63 min, respectively, they were not fully resolved, but could be distinguished based on the appearance of two peak maxima and different absorption maxima (357 nm versus 360 nm, see for chromatographic details Bonin et al.^12^) (Fig. 3). These four MAAs quantitatively dominated in all ocular tissue samples (> 97%). Some chromatograms indicated small traces of unknown compounds, which could represent other MAAs, for example, at retention time 1.37, 1.80, 2.11, and 3.64 min, respectively (Fig. 3). However, identifying them was not possible based on absorption characteristics alone.

Most, but not all samples contained palythine, asterina-330, usujirene and palythene, however, in different quantitative proportions (Fig. 5). Notably, asterina-330 was missing in all eye tissue samples of *Limanda limanda*.

**Fig. 4:**
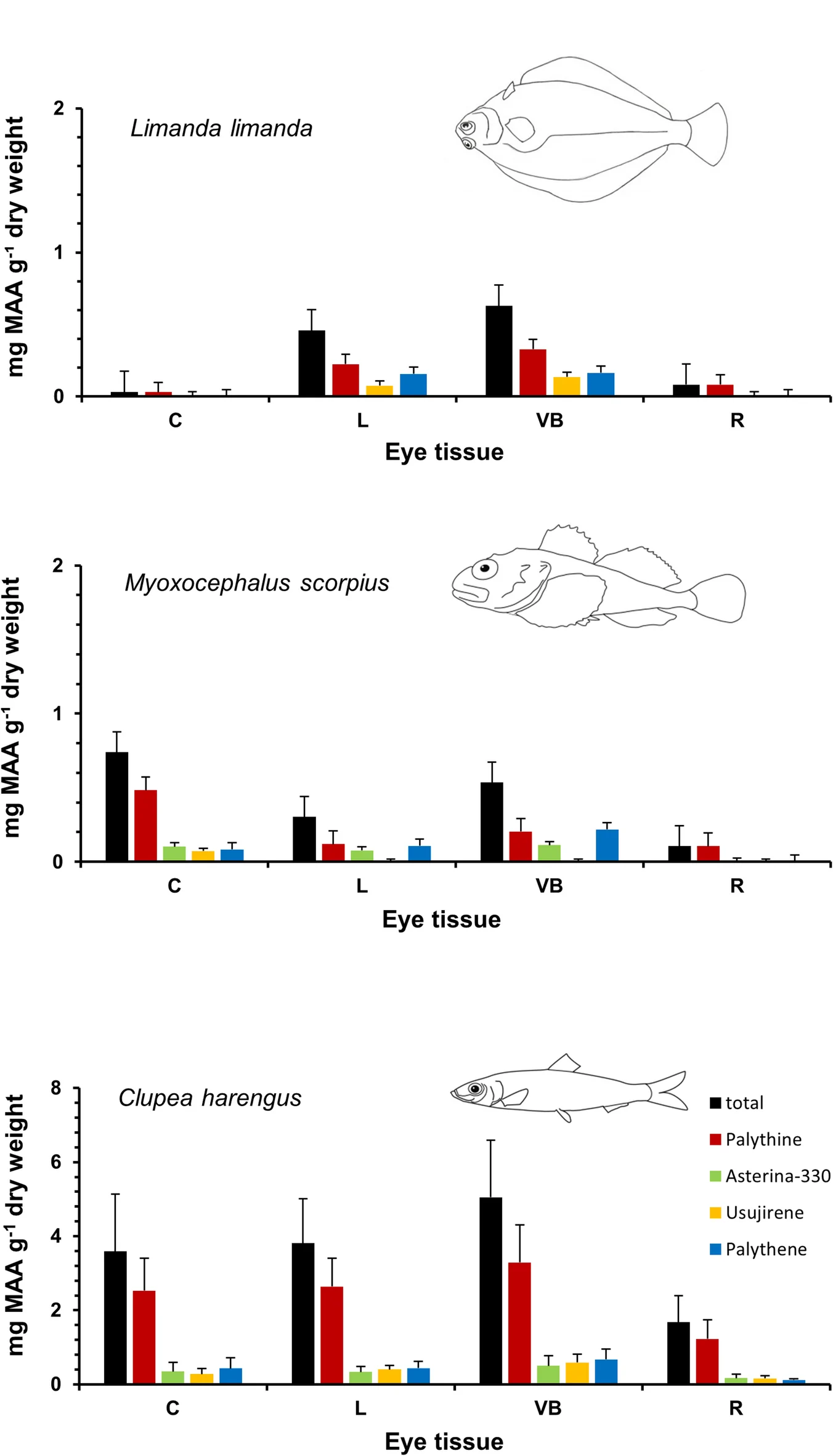
Qualitative and quantitative MAA patterns (mg g^− 1^ dry weight (DW)) in different ocular tissues of the eyes of dab (*Limanda limanda*), sculpin (*Myoxocephalus scorpius*) and herring (*Clupea harengus*; please note the different vertical axis scaling). The eyes were prepared and different tissues dissected: C - cornea (consisting of 3 layers), L - lens, VB – vitreous body and R – retina (plus choroidea and sclera). Values represent mean values ± standard deviation (*n* = 6 for each ocular tissue in *L. limanda*, *n* = 5 for each ocular tissue in *M. scorpius*, *n* = 7 for each ocular tissue in *C. harengus*). Differences between the total MAA concentrations were statistically tested and significantly different (*p* < 0.05) (see also results).

The highest total MAA concentrations were found in *Clupea harengus*. Cornea, lens and vitreous body contained 3.59 ± 2.54 mg g^− 1^ dry weight (DW), 3.81 ± 1.2 mg g^− 1^ DW, and 5.05 ± 2.79 mg g^− 1^ DW), respectively, while the innermost RCS layer showed a smaller content of 1.68 ± 0.71 mg g^− 1^ DW (Fig. 4). The dominant MAA in all samples of *C. harengus* was palythine, representing 65.1 to 73.2% of the total amount. The remaining MAAs occurred in almost equal proportions (Fig. 4).

**Fig. 5:**
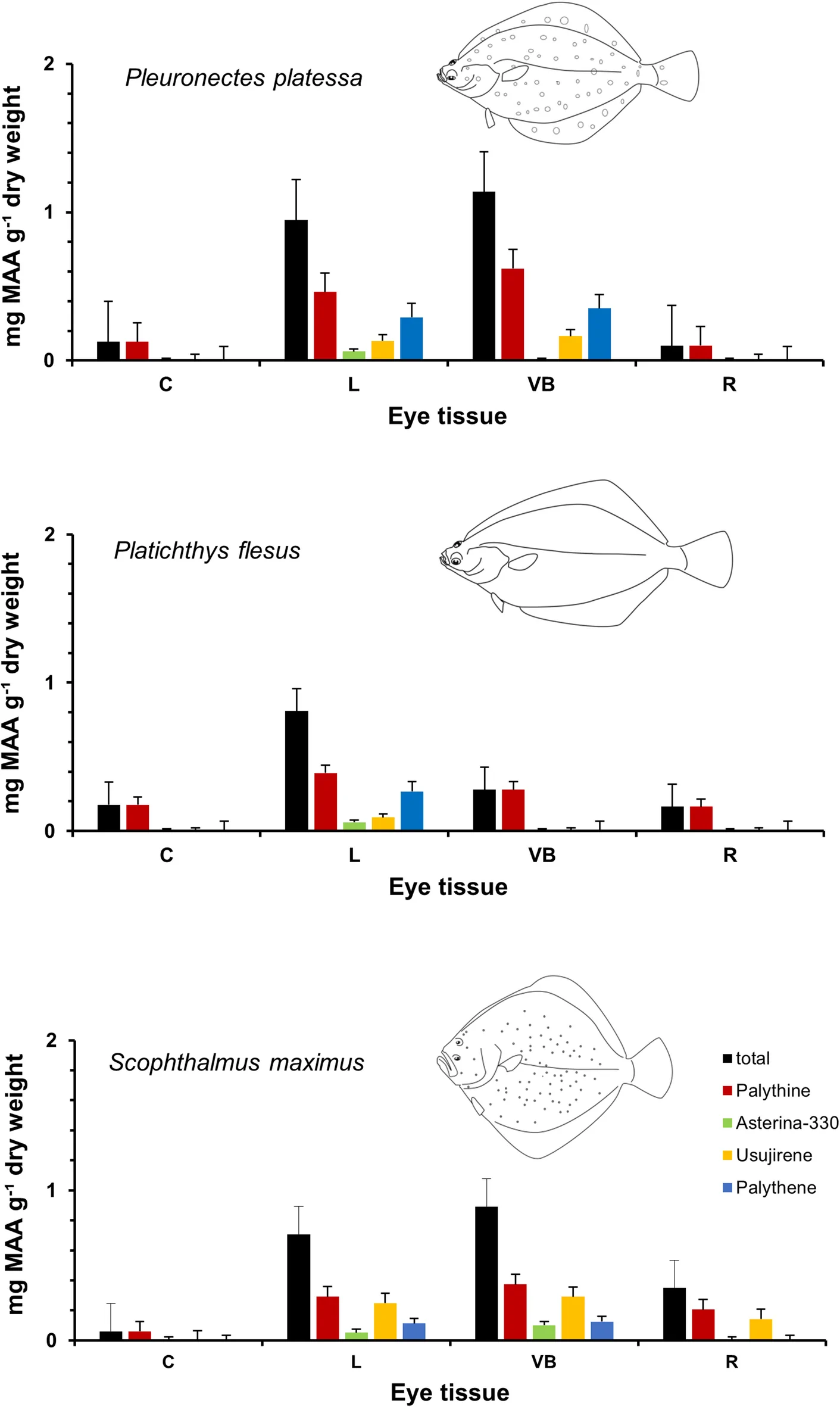
Qualitative and quantitative MAA patterns (mg g^− 1^ dry weight (DW)) in different ocular tissues of the eyes of European plaice (*Pleuronectes platessa*), European flounder (*Platichthys flesus*) and turbot (*Scophthalmus maximus*). The eyes were prepared and different tissues dissected: C - cornea (consisting of 3 layers), L - lens, VB – vitreous body and R – retina (plus choroidea and sclera). Values represent mean values ± standard deviation (*n* = 5 for each ocular tissue in *P. platessa*, *n* = 6 for each ocular tissue in *P. flesus*, *n* = 5 for each ocular tissue in *S. maximus*). Differences between the total MAA concentrations were statistically tested and significantly different (*p* < 0.05) (see also results).

The quantitative sum of all MAAs per sample were much lower in the other five fish species, ranging from 0.03 ± 0.02 mg g^− 1^ DW to 1.13 ± 0.33 mg g^− 1^ DW (Figs. 4 and 5). In *L. limanda* and *S. maximus* the cornea contained only traces of MAAs, mainly palythine. The cornea of *Platichthys flesus* and *P. platessa* exhibited also palythine (0.13 to 0.18 mg g^− 1^ DW, respectively). In the cornea of *M. scorpius* 0.73 ± 0.58 mg palythine g^− 1^ DW were determined (Figs. 4 and 5). The lens and vitreous body of *L. limanda*, *P. platessa* and *S. maximus* showed very similar total MAA concentrations (lens: 0.47–0.94 mg g^− 1^ DW; vitreous body: 0.63–1.13 mg g^− 1^ DW), while in *M. scorpius* the total amount in the lens was only 0.31 mg g^− 1^ DW. In *P. flesus* total MAA concentrations were almost 3-times higher in the vitreous body than in the lens (Figs. 4 and 5). The total MAA contents of the RCS in *P. flesus*, *P. platessa*, and *L. limanda* were consistently low (ranging between 0.08 and 0.16 mg g^− 1^ DW) and comparable to levels measured in the cornea. This means, in most species the lens and vitreous body showed the highest MAA levels (Figs. 4 and 5). Differences between the total MAA concentrations were statistically tested: dab (chi-squared = 15.777, df = 3, *p* = 0.0013); sculpin (chi-squared = 8.529, df = 3, *p* = 0.0363); herring (chi-squared = 7.812, df = 3, *p* = 0.05); European plaice (chi-squared = 9.726, df = 3, *p* = 0.022); European flounder (chi-squared = 9.409, df = 3, *p* = 0.0243); turbot (chi-squared = 7.821, df = 3, *p* = 0.0499).

The eyes of the piscivorous predators, including cormorant, harbor porpoise, harbor seal, grey seal, racoon and otter, did not contain any UV-absorbing compounds (data not shown) or amounts were below the detection limit of the employed analytical setup. Therefore, MAAs were most probably not present in these eyes.

## Discussion

### Methodological contributions to the anatomical structure of fish eyes

The general structure of most teleost fish eyes is anatomically quite similar to that of terrestrial vertebrates^26,27^, but their morphological traits seem to be highly diverse between species (e.g. position of the eyes, pupil shape, their size, retinal structure etc.)^28^ and have evolved to meet the specific visual requirements in relation to the diverse aquatic environments used by fishes^28^.

Therefore, in the here investigated fish specimens, 7 T MRI provided geometrically faithful, artifact-free depictions of the whole anterior segment without refraction- or curvature-related distortions, which is advantageous for true-to-scale morphometry and cross-specimen comparability, and hence an essential information for the manual dissection of different ocular tissues. Anterior-segment OCT delivered micrometer-scale detail of corneal layers and the anterior lens capsule, but over a comparatively small field of view and depth range, so coverage depended on targeted scan placement. Taken together, MRI supplies undistorted global context for consistent anatomical referencing, while OCT supplies layer-specific microstructure at the regions of interest. This multimodal strategy underpins our downstream analyses by (i) co-registering sampling sites with high geometric fidelity, (ii) reducing sampling bias when relating local optical/biochemical readouts to ocular anatomy of fish eyes, and (iii) strengthening cross-species comparisons of anterior-segment features relevant to our MAA localization framework.

The cornea of fish eyes represents a very weak positive lens because it has approximately the same refractive index as water^29^. From an ecological standpoint, a major problem of aquatic habitats is the short viewing distances for fishes, which is related to the reduced optical properties of water, as described in the introduction^5^. Consequently, under natural conditions many fish species experience already very limited viewing distances. UVR can act as an additional stressor affecting the visual system of fish eyes.

### UVR effects on fishes and protective mechanisms

Fish exposed to enhanced UVR suffer fatal “sunburn"^30^, which was later histologically characterized as dorsal skin lesions or necrotic areas and congestion of the fins^31–33^. Many fish species can also experience corneal damage as a result of enhanced UVR^34,35^. Cullen and Montith-McMaster (1993)^34^ provided scanning electron microscopical data on rainbow trout (*Oncorhynchus mykiss*) eyes after UVR treatment that indicated anterior subcapsular epithelial cell damage, leading to cataracts, along with lens fiber swelling and eventual rupture. This cell damage resulted in areas of lower refractive index in the anterior subcapsular region of the lens^34^, with negative effects on the vision of the predatory rainbow trout. To the best of our knowledge the presence of MAAs in the eyes of rainbow trout has not been explored, but in closely related salmonids such as *Salmo salar* and *S. trutta* MAAs are lacking^12^, which well explains the UV-induced ocular tissue damage described before^34^.

Although UVR has multiple negative effects fishes can use various protective strategies to escape this biologically harmful radiation. UVR avoidance behaviors seem to be of particular importance. This includes, for example, physical protection by laying eggs inside gravel of the river bed, spawning inside macrophyte vegetation belts or deeper in the water column^36–38^. Seeking shaded places or greater depths are typical UVR avoidance strategies^8,9^, that require UV photoreceptors for vision or for negative phototactic responses, and, indeed, these have been reported in various fish species^21,39,40^. However, if one or the other avoidance strategy fails and damage to essential biomolecules (DNA, proteins etc.) by UVR takes place, repair mechanisms are up-regulated^4,6^.

To the best of our knowledge, the eyes of fish represent the quantitatively dominant organ for MAA accumulation^12^, although other organs also exhibit MAAs, but in much lower concentrations^18^. Except Thorpe et al. (1993)^21^ who focused on the occurrence of MAAs mainly in the lens of fish eyes, the few other investigations did not distinguish between different tissue types in such an organ.

### Differential MAA accumulation across ocular tissues in fish

In the present study we undertook for the first time microdissections on eyes of various fish species to prepare different ocular tissues for qualitative and quantitative MAA analysis. The data clearly indicate that MAAs occur in all investigated eye tissues of the selected fish species, but in different quantitative proportions. Most of the individual tissues contained 4 different MAAs, namely palythine, asterina-330, usujirene and palythene, a finding that is in accordance with^12^. Our data also confirm that the eyes of *Clupea harengus* consistently contained one of the highest total MAA contents^12^. In herring, the cornea, lens and vitreous body showed the largest MAA levels, while the innermost RCS layer exhibited a much smaller amount. In contrast, the eye tissues of the other five fish species *Platichthys flesus*, *Pleuronectes platessa*, *Scophthalmus maximus*, *Limanda limanda* and *Myoxocephalus scorpius* contained much lower total MAA values. The four flatfish species exhibited remarkably low interspecific variation in total MAA concentrations compared with the remaining species. Flatfish show during larval development an endocrine-mediated metamorphosis where one eye migrates to the other side of their head^41,42^. Whether this rather unique metamorphosis which profoundly affects eye development and adaptation to low-light and UVR environments might contribute to the observed MAA pattern remains an open question. Nevertheless, besides *C. harengus* also *M. scorpius* exhibited enhanced total MAA concentrations in the cornea, while the remaining fish species showed the highest MAA levels in the lens and vitreous body. The RCS tissue layer was always poor in MAAs.

All these data suggest that species-specific MAA distribution patterns exist within fish eyes. Despite the fact that all the investigated eye tissues contain a rather similar MAA composition, they are mainly located in the cornea and/or lens and vitreous body. A preferential localization of MAAs in one of these tissues will result in an improved photoprotection of the RCS layer, and in particular of the innermost, UVR-sensitive retina. The three MAAs palythine, asterina-330, and palythene exhibit similarly high molar extinction coefficients (Ԑ = 36,200 to 43,800 M^− 1^ cm^− 1^)^2^, but different absorption maxima at 320, 330 and 360 nm, respectively. This combination of MAAs leads to absorbing a broad range of incident UVR-wavelengths. Another mechanistic aspect is the observation that MAAs need water molecules in the direct environment as some kind of anchor (H-bonds) while the rest of MAA molecule rotates (“button on a string” mechanism)^43^, which results in ultrafast heat dissipation of the previously absorbed UVR. While the cornea is directly exposed to the external aqueous milieu, the vitreous body contains a high water content, and hence both ocular tissues well support the suggested physico-chemical “button on a string” mechanism.

The conspicuous MAA distribution pattern between the different fish eye tissues implies a metabolic capacity to position these sunscreen compounds in specific cell layers. Given the trophic transfer of MAAs from the primary producers via zooplankton to the fish^12^, we can only speculate on the underlying biochemical and molecular mechanisms, which are completely unexplored. Numerous fish species have the capacity to take up MAAs from the diet without being degraded during digestion or by digestive fluids, and to internally transfer these sunscreen compounds to the target organ, the eye, where they are deposited. In a recent paper Hammerle et al. (2025)^18^ reported the occurrence of MAAs also mainly in the eyes of three flatfish species (*Platichthys flesus*, *Pleuronectes platessa* and *Scophthalmus maximus*), but additionally in gills, heart, intestine, kidneys, liver, skin, and stomach, albeit in significantly lower concentrations. These authors^18^ speculated that the internal fish organs possibly act as transfer points from the digestive tract to UV-sensitive tissues.

Higher trophic levels such as piscine birds and mammalians obviously lack such mechanisms. The here reported data on eyes of fish-eating predators (i.e., cormorant, harbor porpoise, harbor seal, grey seal, racoon and otter) indicate that MAAs were not detectable in any of these samples. To understand, why fish and the investigated piscine predators conspicuously differ in their biochemical capability to resorb MAAs from the food source and internally transfer and deposit these sunscreen compounds in the eyes, more experimental studies are required. At this stage we can only speculate that aspects such as the quality or strength of digestive fluids, cold-blooded (fish) versus warm-blooded (birds, mammalians) physiology, or existing/missing receptors for MAAs might play a role.

## Conclusion

In conclusion, current evidence suggests that MAAs are efficiently transferred from primary producers via zooplankton to fish, yet this transfer seems to terminate at this trophic level. A further trophic transfer to the eyes of piscine predators apparently does not exist. In fish, a complex but unexplored biochemical/molecular mechanism must exist that mediates the internal transport of MAAs from the digestion organs to the eyes, and here to specific ocular tissues.

Although the Baltic Sea fish species under investigation are mostly demersal, they exhibit a preference for shallow waters that are temporarily exposed to high UVR stress from spring to autumn. All of them depend on vision for successful predation; therefore, their eyes require photoprotection against harmful wavelengths to prevent any cell or tissue damage. MAAs have this UV-sunscreening ability.

However, the Baltic Sea is currently experiencing drastic environmental changes due to various anthropogenic stressors, such as warming and eutrophication. In addition, coastal darkening, driven by terrestrial runoff (humic substances) and increased rainfall, has been identified as another potential stressor for many marine organisms. This reduces the penetration of solar light, including UVR, into the water column. While this may benefit fish species in terms of potential UVR damage, it may have broader ecological implications, including impaired visually mediated foraging or predation.

## Supplementary Information

Below is the link to the electronic supplementary material.

Supplementary Material 1

Supplementary Material 2

## Data Availability

All data supporting the findings of this study are available within the paper. The related raw and meta datasets generated during the current study are available from the corresponding author upon reasonable request.
