# Supplementary material for: UV-protective sunscreen compounds and their differential accumulation across ocular tissues in fish eyes from Baltic Sea
Source: Sci Rep. 2026 Aug 27;16:26898. doi: 10.1038/s41598-026-68431-8 (PMC13518351; doi:10.1038/s41598-026-68431-8)
Supplement: Supplementary file 1 [file 41598_2026_68431_MOESM2_ESM.docx]

Supplementary figure legend

Fig. S1 Representative HPLC chromatograms of methanolic extracts of known MAAs from different biological sources: palythine (eyes of *Sprattus sprattus*, retention time of 1.54 min), asterina-330 (eyes of *Plectropomus leopardus*, retention time of 1.63 min), mycosporine-glycine (marine lichen *Lichina pygmaea*, retention time of 2.07 min) and porphyra-334 (red alga *Porphyra umbilicalis*, retention time of 2.31 min). The lowest chromatogram shows a mix of all four MAAs-.The diode-array detector was set at 330 nm (range: 280-400 nm) and the flow rate of the eluent was 0.5 mL min^-1^ (for analytical details and settings see Material and Methods).
